# Depressive symptoms and web user experience

**DOI:** 10.7717/peerj.4439

**Published:** 2018-02-28

**Authors:** Meinald T. Thielsch, Carolin Thielsch

**Affiliations:** Department of Psychology, University of Münster, Münster, Germany

**Keywords:** Depression, Usability, Content, Website evaluation, Aesthetics, Intention to revisit

## Abstract

**Background:**

Depression, as one of the most prevalent mental disorders, is expected to become a leading cause of disability. While evidence-based treatments are not always easily accessible, Internet-based information and self-help appears as a promising approach to improve the strained supply situation by avoiding barriers of traditional offline treatment. User experience in the domain of mental problems therefore emerges as an important research topic. The aim of our study is to investigate the impact of depressive symptoms on subjective and objective measures of web user experience.

**Method:**

In this two-part online study (*N*_total_ = 721) we investigate the relationship between depressive symptoms of web users and basic website characteristics (i.e., content, subjective and objective usability, aesthetics). Participants completed search and memory tasks on different fully-functional websites. In addition, they were asked to evaluate the given websites with standardized measures and were screened for symptoms of depression using the PHQ-9. We used structural equation modeling (SEM) to determine whether depression severity affects users’ perception of and performance in using information websites.

**Results:**

We found significant associations between depressive symptoms and subjective user experience, specifically of website content, usability, and aesthetics, as well as an effect of content perception on the overall appraisal of a website in terms of the intention to visit it again. Small yet significant negative effects of depression severity on all named subjective website evaluations were revealed, leading to an indirect negative effect on the intention to revisit a website via impaired content perceptions. However, objective task performance was not influenced by depressiveness of users.

**Discussion:**

Depression emerges as capable of altering the subjective perception of a website to some extend with respect to the main features content, usability, and aesthetics. The user experience of a website is crucial, especially as it facilitates revisiting a website and thus might be relevant in avoiding drop-out in online interventions. Thus, the biased impression of persons affected by symptoms of depression and resulting needs of those users should be considered when designing and evaluating E-(Mental)-Health-platforms. The high prevalence of some mental disorders such as depression in the general population stresses the need for further investigations of the found effects.

## Introduction

Innumerable websites are being built to entertain, to inform, or to sell—and countless users access those websites. In the last two decades, this interaction between website and web user has become an important field of research. Thus, the evaluation of websites and their features has been intensively investigated (for overviews see Thielsch & Hirschfeld, in press; Hornbæk, 2006; Moshagen & Thielsch, 2010). However, specific user features might also influence evaluation outcomes and therefore likewise be of interest for human–computer interaction research. Irrespective of whether a website targets the general public or a particular subgroup, it seems necessary to investigate potential characteristics of the groups of people to be addressed. In this regard, we investigate the rather neglected issue of web users with mental problems. We examine whether symptoms of a psychological disorder could affect user experience (UX). More specifically, we focus on the question how people affected by depressive symptoms perceive websites. We chose to investigate this disease as depressive disorders are a quite common mental problem with 12-month prevalence rates between 4% and 11% (Andrade et al., 2000; Kessler & Üstün, 2008; Wittchen & Hoyer, 2011), and are expected to even become a leading cause of disability in the future (Mathers & Loncar, 2006). In Germany, 67% (12-month period) of those who suffer from a depressive disorder remain untreated (Mack et al., 2014). Thus, web-based information and Internet-based treatments are a promising approach to improve the supply situation.

We conducted two studies to explore the impact of depressive symptoms on the appraisal of websites. Subjective (i.e., website evaluations with regard to content, usability and aesthetics) as well as objective measures (i.e., performance in search and memory tasks) were applied. Based on the latter, we examined if performance as an objective measure of usability is altered by depression, i.e., how participants perform in online search and memory tasks depending on the severity of depressive symptoms.

### Perception and evaluation of websites

The perception of a website is primarily constituted by the users’ appraisal of three core website characteristics: content, usability, and aesthetics (e.g., Cober et al., 2003; Tarasewich, Daniel & Griffin, 2001; Thielsch, Blotenberg & Jaron, 2014). For content and usability, standardized definitions are available: In ISO standard 9241–151 (ISO, 2006), the International Organization for Standardization defines content as ‘a set of content objects’ on a web user interface. A content object is an ‘interactive or non-interactive object containing information represented by text, image, video, sound or other types of media’ (ISO, 2006, p. 3). The definition of website usability is often based on ISO 9241-11 as the ‘extent to which a product can be used by specified users to achieve specified goals with effectiveness, efficiency and satisfaction in a specified context of use’ (ISO, 1998, p. 2). However, a differentiation between subjective and objective usability is vital (see Hornbæk, 2006): while a website may be experienced as unusable from a subjective user’s point of view (e.g., caused by misunderstandings or missing functions), it still could perform well on an objective usability measure (e.g., based on fast loading speed or a good search function). A common definition is presently not available for the third main characteristic of a typical website: the design in terms of aesthetics. In research, aesthetics is often described as an immediate pleasurable subjective experience (Leder et al., 2004; Moshagen & Thielsch, 2010; Reber, Schwarz & Winkielman, 2004). Aesthetic perceptions and responses occur immediately at first sight; thus, the visual aesthetics of a website is processed very quickly, often within a split second (e.g., Bölte et al., 2017; Lindgaard et al., 2006; Thielsch & Hirschfeld, 2012).

Those three main characteristics interact in the perception of a website and the use process. Several studies investigate pre- and post-use differences in user evaluations, an excellent overview can be found in Lee & Koubek (2012). They proposed a model in which usability and aesthetics are strongly connected in pre-use evaluations, and relatively weakly connected after use. In the present study, we focus on post-use evaluations of content, usability and aesthetics. In addition, we investigate an outcome variable with special importance for practice: the overall appraisal of a website in terms of the intention to visit a given website again. The three main website characteristics contribute to this overall appraisal of a website (e.g., Kang & Kim, 2006; Lee & Koubek, 2012; Moshagen & Thielsch, 2010). Previously, using structural modeling of the data in two different studies, Thielsch and colleagues (2014) found that content has the largest influence on the user’s intention to revisit a website, while only a small effect was found for aesthetics, and subjective usability did not exhibit a significant influence. However, two aspects were not investigated in these studies: (a) objective usability and (b) user characteristics.

User characteristics are part of widespread user experience models, such as the CUE model (“Components of User Experience”, Thüring & Mahlke, 2007), as antecedents of an interaction experience with a website or a technical system. Thus, according to the CUE model, user characteristics are indirectly responsible for user experience outcomes such as an overall impression, and the revisitation of a website. However, there are only few UX studies systematically investigating user characteristics: for example, the effects of age (e.g., Sonderegger, Schmutz & Sauer, 2016; Thielsch, 2008), gender (e.g., Bardzell & Churchill, 2011; Tuch, Bargas-Avila & Opwis, 2010) or personality (e.g., Bosnjak, Galesic & Tuten, 2007; Burnett & Ditsikas, 2006; Thielsch, 2008). Only a few studies have been published with a focus on the web user experience of individuals with mental disorders. Due to the high prevalence of depression in the population, we aim to specifically investigate web users with different levels of depression severity.

### Depressive disorders

Depressive disorders typically involve mood impairment and rank among the most frequent mental disorders throughout the world. Comparing samples from the USA, Canada, Germany, Netherlands, Mexico, Brazil, and Turkey, Andrade and colleagues (2000) found 30-day prevalence rates from 2% up to 5%, 12-month prevalence rates between 4% and 11%, as well as lifetime prevalence rates between 7% and 19%. Despite an increasing use of antidepressants over the last two decades, evidence that prevalence rates decline over time could not yet be found (Patten et al., 2016). Thus, a certain number of participants can be expected to be affected by symptoms of depression even in a common website test using a convenience sample or a usual panel sample.

Major Depressive Disorder (MDD) is part of the depressive disorders and describes a fully developed mood disorder. In addition to depressed or irritable mood, MDD is also characterized by decreased interest or pleasure in most activities, significant changes in weight or appetite, sleep, and activity, as well as psychomotor agitation or retardation, fatigue or loss of energy, feelings of guilt or worthlessness, impaired concentration, and suicidal ideation (American Psychiatric Association, 2013). In addition to these various symptoms, Blanco et al. (2014) present evidence for high comorbidity rates: the prevalence of at least one comorbid psychiatric disorder was 76% among patients with MDD. In addition to MDD, 54% were diagnosed with a substance use disorder, 31% with an anxiety disorder, 16% revealed another mood disorder, 28% a personality and 1% a psychotic disorder.

To illustrate the suffering of the people affected, Murray et al. (2012) report an increase in *disability-adjusted life years* (DALYs as the sum of *years of life lost* (YLLs) and *years lived with disability* (YLDs); see Murray & Lopez, 1996) which made major depressive disorder climb to place 11 in causing the most DALYs of 291 diseases in 2010 (compared to place 15 in 1990). More specifically, MDD causes impairment in self-care, mobility, cognition, social functioning and role functioning (Kessler et al., 2003). In addition to the suffering, direct and indirect costs to society are caused as well: mood disorders impair work performance. Kessler et al. (2006) estimate 27.2 annually lost workdays per ill worker with an associated annual capital loss of $4426.

Summing up, investigating depressive symptoms and their effects in everyday life is not only justified by the frequency of its occurrence, but also by the far-reaching consequences they entail. Affecting mood, cognition and behaviour, depressive symptoms are suspected to globally alter peoples’ perceptions—consequently also with regard to web user experience. In the evaluation of website-features, depressive mood, an overall loss of interest, difficulties to concentrate on the specific content (see diagnostic criteria, American Psychiatric Association, 2013 and Harvey, 2007) as well as biased evaluation patterns sensu Beck (i.e., Beck et al., 1979) might serve as specific mediatory processes. Altered web search-performance might also be driven by impaired cognitive performance and by psychomotor anomalies that are likewise listed among the diagnostic criteria (see American Psychiatric Association, 2013).

### E-Mental-Health, depression and user experience

In Germany, 67% (12-month period) respectively 44% (lifetime) of individuals who suffer from a depressive disorder remain untreated—and this proportion may be relatively low by international standards (Mack et al., 2014). Furthermore, a large proportion of affected people that do receive treatment, do so with a delay which is possibly aggravating their problems (Wang et al., 2007; Wittchen et al., 2011).

The use of Internet-based treatment (“E-health” or more specifically “E-mental-health”), such as online-platforms offering information and/or aiming at reducing depressive symptoms, seems to be an important approach to improve the supply situation of people with depression. It is aimed at overcoming limitations of traditional treatment services by increasing treatment availability and reducing barriers of access (Ebert et al., 2015). Whether online-based interventions also entail burdens due to features of Internet-use, such as a lack of personal contact, is a relatively new research topic. Knowles and colleagues (2014) performed a review of eight qualitative studies on computerized depression (and anxiety) treatments: they revealed that advantages of such therapies, such as more privacy and higher experienced control, could also be perceived as limitations compared to classical face-to-face interventions. Additional studies confirmed this conclusion, demonstrating that patients with depressive disorders had mixed experiences with computerized treatments (Gega, Smith & Reynolds, 2013; Knowles et al., 2015). Yet Internet-based treatments of depression were comparable in terms of drop-out (Christensen, Griffiths & Farrer, 2009), and proved to be effective and lead to general satisfaction of participants (Davies, Morriss & Glazebrook, 2014; Richards & Richardson, 2012). Nevertheless, the research on user experiences in depressed web-users as a whole is still in its infancy.

Few studies have investigated the usability of specific interventions (e.g., Kasckow et al., 2014; Tiburcio et al., 2016) or user experience of specific online platforms and websites designed for people suffering from depressive disorders (Morris, Schueller & Picard, 2015; Prusti et al., 2012). The lack of literature emphasizes the need for a differentiated investigation of the interaction between depression and user experience, respectively between web users with depressive symptoms and information websites.

### Research question and hypotheses

**Figure 1:**
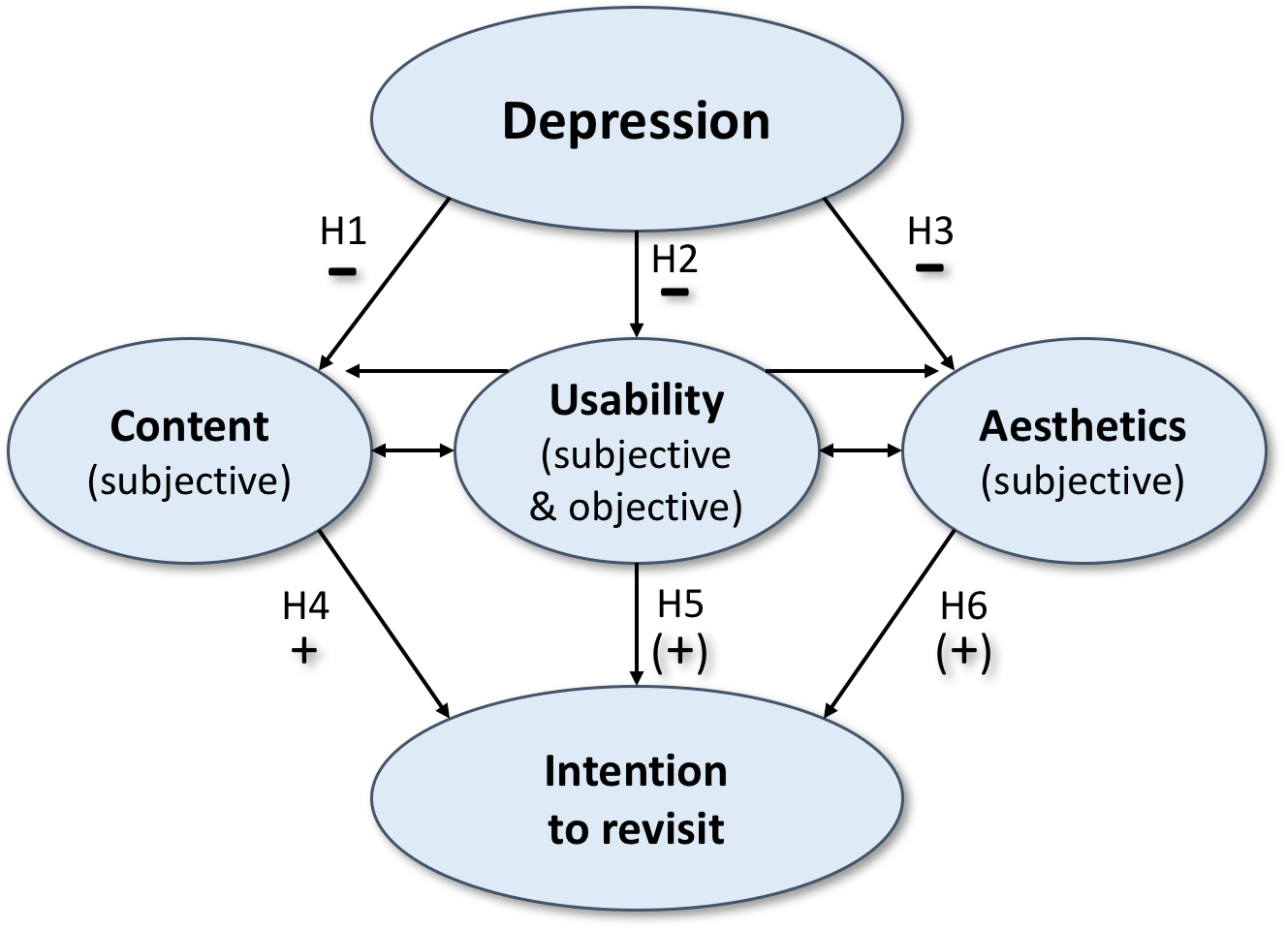
The hypothesized model; single-headed arrows represent the impact of one variable on another, while double-headed arrows represent co-variances between pairs of the latent variables.

In this paper, we investigate the impact of depression on subjective as well as objective measures of web user experience. Subjective measures refer to an evaluation of three main website characteristics (i.e., content, subjective usability, and aesthetics), while objective measures capture performance rates in online search and memory tasks (i.e., objective usability). In both cases, we expect impaired results due to depressive symptoms: worse evaluations of all three subjective main website characteristics as well as poorer outcome in objective performance-tasks. The hypothesized model (Fig. 1) was established by integrating depression severity as the user characteristic of interest in the model of web user evaluation proposed by Thielsch and colleagues (2014). Depression is seen as a disease located on a continuum from healthy to sick, thus treated as continuously variable. As argued above, depression is expected to decrease subjective and possibly even objective measures of content, usability and aesthetics, which again potentially increase the intention to revisit. Based on previous research (Thielsch, Blotenberg & Jaron, 2014) the overall appraisal of a website in terms of the intention to revisit should be highly influenced by content perceptions, while the impact of perceived usability or aesthetics is expected to be weaker. Yet as prior research in this context has not investigated objective usability, we aim to further explore this matter.

 The specific study hypotheses are:

H1 (depression → content): Depression negatively influences the subjective appraisal of website content.
H2a (depression → subjective usability): Depression negatively influences the subjective appraisal of website usability.
H2b (depression → objective usability): Depression negatively influences objective measures of website usability.
H3 (depression → aesthetics): Depression negatively influences the subjective appraisal of website aesthetics.
H4 (content → intention to revisit): Subjective appraisal of website content positively influences the intention to revisit.
H5a (subjective usability → intention to revisit): Subjective appraisal of website usability marginally positively influences the intention to revisit.
H5b (objective usability → intention to revisit): Objective measures of website usability marginally positively influence the intention to revisit.
H6 (aesthetics → intention to revisit): Subjective appraisal of website aesthetics marginally positively influences the intention to revisit.

A two-study approach was used to test the robustness of the findings, as Study 2 aims at replicating and extending the findings deriving from Study 1.

## Study 1

### Method

#### Participants

Participants were recruited online (via the online-panel PsyWeb https://psyweb.uni-muenster.de, social networks, relevant thematic forums and mailing lists) and offline (via newspaper advertisements and leaflets). The study was announced as a psychological research study including a feedback about depressiveness.

A total of *N* = 618 individuals started to participate in the web-based study, *n* = 344 finished it and *n* = 333 released their data—six participants had to be excluded due to age limits and very short processing time. The final sample consisted of *n* = 329 web users (65% female), age ranged from 16 to 88 years (*M* = 30.8; SD = 11.3). The participants on average spent 14.78 h per week surfing the Internet (SD = 10.34). With respect to depression severity, the sample included the full range of possible answers in the PHQ-9: There were participants who reported no symptoms at all and others who stated to experience a severe level of depression.^1^ 1In the present research, depression severity is analysed as a continuous variable. Thus, we did not use diagnostic categories. If diagnostic cut points are applied as given in the PHQ-9 manual, a subsample of *n* = 113 would be classified as low (PHQ-9 score < 5), *n* = 129 as moderate (PHQ-9 score between 5 and 10) and *n* = 87 as high on depression severity (PHQ-9 score > 10).Participants took part voluntarily and anonymously without any compensation.

#### Measures

Scales for website evaluation were the same as in Study 3 of Thielsch, Blotenberg & Jaron (2014)—except for content, here a revised version of the according questionnaire was used (see Thielsch & Hirschfeld, in press). All subjective website evaluation scales were rated on a 7-point Likert scale, ranging from “1—strongly disagree” to “7 –strongly agree”. The following questionnaires were applied to measure depression and to evaluate the website:

##### Depression.

The Patient Health Questionnaire (PHQ; Spitzer, Kroenke & Williams, 1999); (German version: Gräfe et al., 2004) is based on the diagnostic criteria from the DSM-IV. We used the 9-item depression subscale (e.g., “Little interest or pleasure in doing things.”; “Trouble concentrating on things, such as reading the newspaper or watching television.”). The items have to be rated on a 4-point Likert scale (0 = “not at all”, 1 = “several days”, 2 = “more than half the days”, 3 = “nearly every day”) with a total sum-score capturing overall depression to be calculated. Good psychometric properties (*α* in this sample = .89) have been reported (Gräfe et al., 2004).

##### Website content.

To measure subjective website content perception the Web-CLIC (Website—Clarity, Likeability, Informativeness, and Credibility; Thielsch & Hirschfeld, in press) was used. This questionnaire consists of twelve items on four subscales representing a general factor ‘subjective perception of content’ (example items: “The website is informative.”, “The texts provide me information in a clear and concise manner.”, see Table A.1). The questionnaire has been shown to possess good psychometric properties in terms of high reliability, stability, as well as convergent, divergent, discriminative, concurrent and experimental validity (see Thielsch & Hirschfeld, in press). A sum score reflecting the average value is computed; in this sample, *α* = .95.

##### Website usability.

To measure *subjective usability*, a scale adapted to German from Flavián, Guinalíu & Gurrea (2006, see Thielsch, 2008; Thielsch, Engel & Hirschfeld, 2015) was used that consists of seven items (example items: “This website is simple to use, even when using it for the first time.”, “It is easy to navigate within this website.”, see Table A.2). The usability scale shows factorial validity and high internal consistency (Thielsch, 2008; *α* = .94 in this sample).

To operationalize *objective usability*, several search-tasks referring to the presented information were displayed (see Hornbæk, 2006). Additionally, after time interval of approximately ten minutes between presentation and testing, a recall of the reading matter was tested. Memory tasks were used as typical measure of usability in terms of effectiveness (see Hornbæk, 2006). A multiple-choice format with four optional answers each was presented to collect the answers while minimizing guess probability. Correct answers in the search-tasks (two tasks per website, for example: “How many patients profit from antidepressants in the first two weeks of treatment?”) and five memory tasks (referring to the main website, for example: “What percentage of people older than 65 years are suffering from depression?”) were used to generate a total score (range 0–7) to be entered into further analyses as an objective measurement of usability. At the same time, the assignment of tasks allowed to check whether the participants actually dealt with the presented websites.

##### Website aesthetics.

The short version of the *VisAWI* (Visual Aesthetics of Websites Inventory; Moshagen & Thielsch, 2010; Moshagen & Thielsch, 2013) was used to capture an overall rating of a website’s aesthetic impression. The VisAWI-S contains four items (example items: “The colour composition is attractive.”, “The layout is pleasantly varied.”, see Table A.3) and shows good reliability and construct validity in terms of convergent, divergent, and concurrent validity (see Moshagen & Thielsch, 2013). In this sample, *α* = .88.

##### Intention to revisit.

The intention to revisit as an overall measure of appreciation, describing a relevant behaviour in online self-help, was captured using three items (“I will visit the website again.”, “I will visit the website on a regular basis.”, “If I had interest in the content of the website in future, I would consider visiting the website again.”). They were taken from Moshagen & Thielsch (2010) and aggregated as proposed by Thielsch, Blotenberg & Jaron (2014, p. 97). In this sample, *α* = .84.

#### Procedure and stimulus material

At the beginning of the study, participants provided demographic information and health details regarding depressive symptoms (via the PHQ-9).

Afterwards, they completed online search-tasks (see Table A.4) on two fully functioning health-related websites that were randomly displayed. One of the tested websites was the same for all participants (“main site” presenting information about depressive disorders, these data were entered into the main analyses) and the second one was randomly picked out of four websites as a contrast stimulus. The contrast websites were chosen to systematically present (a) comparable content about depression in a different website design, (b) different content in a different website design (presenting information about “physical training”), or (c) different content in the same website design as the main site (information on “physical training” or “health insurance formalities”). Participants evaluated the first presented websites with regard to content, usability, aesthetics, and intention to revisit. Afterwards, memory tasks (see Table A.4) were given; then the procedure was repeated with the second website. The contrast site was used to check if the website design or topic systematically interfered with results. This was not the case, hence we further analysed only evaluations for the main site as it was seen by all participants.

At the end of the study, participants were thanked and given the opportunity to exclude the provided data from further analyses. Feedback regarding depressive symptoms was given and participants could comment on the study. On average, participants needed 34 min to complete the study.

#### Statistical analyses

IBM SPSS Statistics 22.0 (IBM Corporation) was used for descriptive data analysis. After computing correlational relationships, a two-step approach was used to test the hypothetical model using Mplus (Muthén & Muthén, 1998–2010). First, a series of confirmatory analyses (CFA) was conducted to assert acceptable fit of the measures. Second, structural equation modeling (SEM) was performed to test structural relationships among the variables of interest. All variables were group mean centered. Possible distortions due to deviations from a normal distribution respectively through skewness and kurtosis of the variables’ distributions were prevented by Satorra-Bentler scaling the data (see Hu & Bentler, 1999). The goodness of fit was monitored computing several fit indices including the RMSEA, CFI, TLI and SRMR in addition to the chi-square test.

### Results

We first tested whether the variables of interest were significantly related to demographic factors: gender did not reveal significant differences regarding depression (*t* = .35, *p* = .73), website features (content: *t* =  − .76, *p* = .45; usability: *t* =  − .31, *p* = .76; aesthetics: *t* =  − 1.16, *p* = .25) or the intention to revisit (*t* =  − 1.88, *p* = .60). Likewise, the level of education did not portray significant gaps with regard to depression (despite a significant main effect, *F*(4, 324) = 2.56, *p* = .04, post hoc comparisons using Bonferroni correction did not reveal significant differences between the five educational groups, ranging from no school-leaving qualification to higher education entrance qualification), website characteristics (content: *F*(4, 324) = 1.88, *p* = .11; usability: *F*(4, 324) = 1.02, *p* = .40; aesthetics: *F*(4, 324) = .78, *p* = .54) or the intention to revisit (*F*(4, 324) = 2.06, *p* = .09). Finally, age did not correlate with ratings of depression (*r* =  − .03, *p* = .58), content (*r* =  − .05, *p* = .36) or aesthetics (*r* =  − .06, *p* = .26), while it showed a significant relationship with usability (*r* =  − .11, *p* = .04) and the intention to revisit (*r* = .15, *p* < .01).

Table 1 shows means, standard deviations, and intercorrelations between all variables under study. As objective usability was not significantly correlated with depression severity or intention to revisit, it was excluded from further analyses. The remaining variables are correlated as expected: depression shows significant negative relationships with all three website features (content, usability, aesthetics) measured via questionnaires (−.15 ≤ *r*’s ≤ − .13; all *p*’s <.05). Again, according to expectations, the intention to revisit is significantly positively correlated with all three dimensions of web user experience (.39 ≤ *r*’s ≤.69; all *p*’s <.01).

**Table 1:** Study 1: mean, standard deviations and correlations among variables of interest (*N* = 329)—website “depressive disorders”.

Measure	1	2	3	4	5	6
1. Depression (PHQ)	–	−.15^**^	−.13^*^	−.06	−.15^**^	−.03
2. Content (Web-CLIC)		–	.62^**^	.26^**^	.72^**^	.69^**^
3. Usability (subjective)			–	.39^**^	.58^**^	.39^**^
4. Usability (objective)				–	.19^**^	.09
5. Aesthetics (VisAWI)					–	.53^**^
6. Intention to revisit						–
MW	7.67	4.97	4.95	4.21	4.89	3.18
SD	5.49	1.16	1.32	1.41	1.24	1.43
Range	0–27	1–7	1–7	0–7	1–7	1–7
Item loadings	.51–.82	.69–.88	.76–.93	−.04^a^–.76	.78–.85	.73–.98

**Notes.**

* Indicates that a correlation is significant at a level of *p* < 0.05 (two sided).

** Indicates that a correlation is significant at a level of *p* < 0.01 (two sided).

a Deleting the item with poor load or splitting into search and memory tasks did not lead to significant correlations with depression.

PHQ Patient Health Questionnaire Web-CLIC Website—Clarity, Likeability, Informativeness, Credibility VisAWI Visual Aesthetics of Websites Inventory

**Figure 2:**
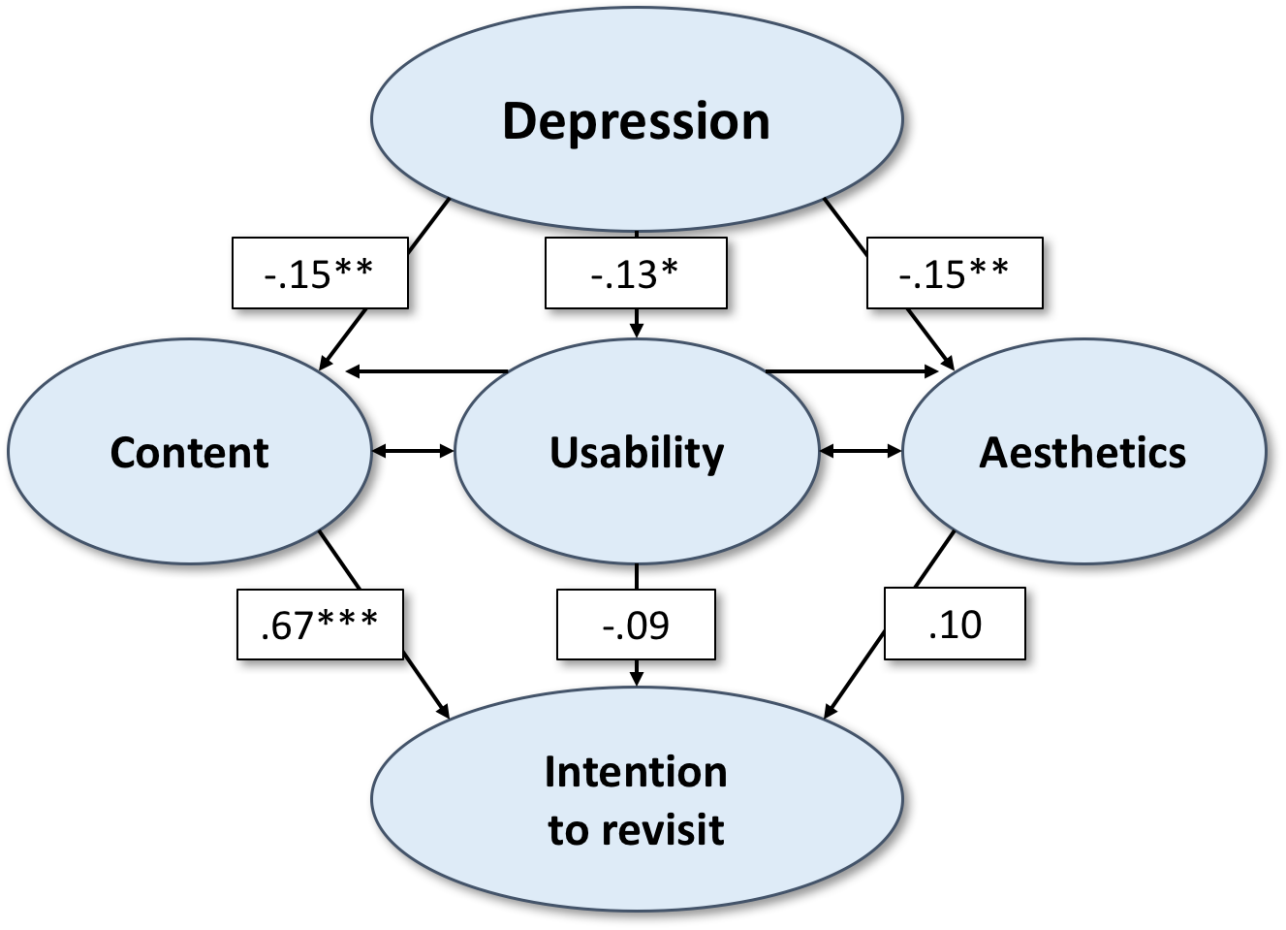
Structural equation model showing significant relationships between the variables of interest in Study 1 (main website presenting information on depression).

Using structural equation modeling, the hypothetical relationships were investigated simultaneously (see Fig. 2; Table 2). All item loadings of the parameters integrated in structural equation modeling were >0.5 (Table 1). The pathway estimates (*β*) are reported in a standardized format. As hypothesized, depression was significantly negatively associated with content, usability and aesthetics (−.15 ≤ *β*’s ≤ − .13; all *p*’s <.05). However, with regard to the website features, only content revealed a significant positive association with the intention to revisit (*β* = .67, *p* < .01).

**Table 2:** Model parameters (standardized).

Regression on/correlation with	Estimate	S.E.	Two-tailed *P*-Value
Content on Depression	−0.15	0.06	<0.01
Usability on Depression	−0.13	0.06	0.02
Aesthetics on Depression	−0.15	0.06	0.01
Intention to Revisit on			
Content	0.67	0.06	<0.01
Usability	−0.09	0.05	0.10
Aesthetics	0.10	0.05	0.06
Content with			
Usability	0.62	0.04	<0.01
Aesthetics	0.71	0.03	<0.01
Usability with Aesthetics	0.58	0.04	<0.01

Overall, the model fitted the data satisfactorily. The chi-square test as a measure of exact fit shows the preferable non-significant result (*chi square* = 3.267, *df* = 1, *p* = .071). The same applies for the additional measures of approximate fit: the RMSEA (=.083) lies between .08 and .10, thus providing a mediocre fit (MacCallum, Browne & Sugawara, 1996). This seems defendable when analysing a model with various parameters leading to restrained fit with regard to the RMSEA. CFI (=.996) and TLI (=.963) exceed the cut-off 0.95 and therefore indicate a good fit (Hu & Bentler, 1999). As for the SRMR, values less than .05 are obtained by well fitting models (in this study SRMR = .017; Byrne, 1998).

### Discussion

Applying the hypothetical model, we find that depression significantly influences all three dimensions of website user experience measured by questionnaire (confirming *H1, H2a, H3*). In line with previous research results (Thielsch, Blotenberg & Jaron, 2014), significant path coefficients leading from website features to the intention to revisit them can be reported for content only (confirming *H4*), while the remaining UX features did not significantly influence this intention (rejecting *H5, H6*).

Furthermore, we did not find significant associations with an objective measurement of usability (rejecting *H2b*). There are several possible explanations: (a) There is no measurable impairment due to depressive symptoms in online performance at all; (b) such a decline is only to expect in severe depression (which is not focused in the present study); (c) the possibility that, even as the tasks used in this study were typical for Internet use, they are too easy to capture impaired performance caused by depression. The differences between individuals low on depression severity compared to those high on depression severity are apparent in the significant path coefficient leading from depression to subjective usability, but possibly could be compensated by the individuals affected when it comes to fulfilling the tasks capturing objective usability. In the latter case (actual impairment could not be detected), the operationalization of objective usability should be revised to once again try to capture impaired performance. Additionally, as data from only one website were collected in the whole sample, a replication of the study appeared to be necessary to test the robustness of the findings.

## Study 2

Study 2 aimed to replicate and extend the findings derived from Study 1. The amount of search and memory tasks was increased to capture a possible impairment of objective usability due to depressive symptoms. Furthermore, two different websites were tested in a within-subject design for comparison: an information website on physical training and a mock-up one that allowed to be fully controlled. We thereby aimed at reducing a possible evaluation bias due to depressive symptoms by using websites dealing with health-related topics other than depression.

### Method

#### Participants

Recruiting of participants was analogous to Study 1. A total of *N* = 586 individuals started to participate in the online study, *n* = 402 finished it and *n* = 394 released their data; yet, data of two participants had to be excluded due to technical problems. The final sample of Study 2 consisted of *n* = 392 web user (60% female), age ranged from 16 to 75 years (*M* = 43.8; SD = 12.4). On average, participants spend 14.90 h per week surfing the Internet (SD = 12.85). With respect to depression severity, the sample included nearly the full range of possible answers in the PHQ-9.^2^ 2Again, depression severity is analysed as a continuous variable and we used no diagnostic categories. If diagnostic cut points are applied as given in the PHQ-9 manual, a subsample of *n* = 147 would be classified as low (PHQ-9 score < 5), *n* = 137 as moderate (PHQ-9 score between 5 and 10) and *n* = 108 as high on depression severity (PHQ-9 score > 10).Again, participants took part voluntarily and anonymously without any compensation.

#### Measures and stimulus material

The same measurements as in Study 1 (see ‘Measures’) were used to capture depression severity, content, usability, aesthetics, and the intention to revisit. As Study 1 failed to reveal the theoretically expected effect of depression on the objective measurement of usability, the operationalization was extended in Study 2: Again, search and memory task were used. Yet this time six search-tasks (three tasks per website, for example: “What percentage of the general population is suffering from glaucoma?”) and eight memory tasks (four tasks per website, for example: “How is arthrosis called in colloquial language?”) were presented. Total scores (range per website: 0–7) of correct answers were entered into further analyses as objective measures of usability.

Additionally, this time the two fully functioning websites were randomly presented to all participants in order to be able to test the robustness of the findings by comparing different website-evaluations in one study. One website was an existing one (A: “Physical training”) giving information on training and health exercises. The other was a mock site with health-related medical and psychological information (B: “MedOnline”, see Appendix A.1), which had been created by an experienced web designer for research purposes of our working group.

#### Procedure and statistical analyses

The procedure was analogous to Study 1: participants provided demographic information as well as depression scores, were randomly assigned to the first of the two websites, completed search tasks (see Appendix A.5), evaluated the website, and answered to the memory tasks (see Appendix A.5). Again, the participation was concluded with feedback and the option of self-exclusion. It took approximately 36 min to complete the study.

The statistical analyses were conducted in the same manner as in Study 1 (see ‘Statistical analyses’).

### Results

As in Study 1, we first searched for significant relationships between variables of interest and demographic factors. Again, gender did not reveal significant group differences regarding website features (content A: *t* =  − .12, *p* = .90, B: *t* =  − .44, *p* = .66; usability A: *t* = 1.06, *p* = .29, B: *t* =  − .22, *p* = .82; aesthetics A: *t* =  − .09, *p* = .93, B: *t* =  − .57, *p* = .57) or the intention to revisit (A: *t* =  − .79, *p* = .43, B: *t* =  − 1.23, *p* = .22), but was significant for depression (*t* = 3.12, *p* < .01; female participants: *M* = 17.45, SD = 6.19; male: *M* = 15.57, SD = 5.61).

Furthermore, the level of education did not differ for depression (*F*(4, 387) = .91, *p* = .46), website characteristics (content A: *F*(4, 387) = 1.05, *p* = .38, B: *F*(4, 387) = 1.43, *p* = .22; usability A: *F*(4, 387) = 1.04, *p* = .39, B: *F*(4, 387) = .94, *p* = .44; aesthetics A: despite a significant main effect, *F*(4, 387) = 2.61, *p* = .04, post hoc comparisons using Bonferroni correction did not reveal significant differences between the five educational groups ranging from no school-leaving qualification to higher education entrance qualification, B: *F*(4, 387) = 2.17, *p* = .07) nor the intention to revisit (A: *F*(4, 387) = .94, *p* = .44, B: *F*(4, 387) = .31, *p* = .87).

Finally, age did not correlate with ratings of content (A: *r* =  − .003, *p* = .96, B: *r* =  − .02, *p* = .71), usability (A: *r* = .01, *p* = .82, B: *r* = .10, *p* > .05), aesthetics (A: *r* =  − .04, *p* = .45, B: *r* =  − .05, *p* = .33), but partly with the intention to revisit (A: *r* = .12, *p* = .02, B: *r* = .08, *p* = .13) and with depression (*r* =  − .20, *p* < 0.01). As male participants (*M* = 47.28; SD = 12.34) were significantly older (*t* =  − 4.69, *p* < .01) than female (*M* = 41.44; SD = 11.82) this relationship between age and depression might partly be mediated by gender (gender → depression: *β* =  − .11, *p* = .03; gender → age: *β* = .23, *p* < .01; age → depression: *β* =  − .18, *p* = .01).

Table 3 (referring to website A: “Physical training”, with information on health exercises) and Table 4 (referring to website B: “MedOnline”, with health information) show means, standard deviations, and intercorrelations between all variables under study. Again, the total score referring to the search and memory tasks (objective measure of usability) did not significantly correlate with depression severity (A: *r* =  − .03; *p* = .62; B: *r* =  − .06, *p* = .27)^3^ 3Likewise, computing two specific scores for the search and memory tasks does not lead to significant correlations with the PHQ.and correspondingly was excluded from further analyses again. However, we did find significant correlations between objective usability and the intention to revisit in both cases (A: *r* = .22, *p* < .01; B: *r* = .19, *p* < .01), possibly indicating an improvement in operationalization.

**Table 3:** Study 2: mean, standard deviations and correlations among variables of interest (*N* = 392)—website A “Physical training”.

Measure	1	2	3	4	5	6
1. Depression (PHQ)	–	−.12^*^	−.15^**^	−.03	−.15^**^	−.05
2. Content (Web-CLIC)		–	.70^**^	.30^**^	.76^**^	.71^**^
3. Usability (subjective)			–	.35^**^	.70^**^	.48^**^
4. Usability (objective)				–	.21^**^	.22^**^
5. Aesthetics (VisAWI)					–	.54^**^
6. Intention to revisit						–
MW	7.70	4.75	4.40	2.85	4.84	3.26
SD	6.03	1.26	1.44	1.40	1.35	1.62
Range	0–27	1–7	1–7	0–7	1–7	1–7
Item load	.58–.88	.68–.90	.80–.93	.09^a^–.55	.77–.90	.80–.98
Cronbach’s Alpha	.90	.96	.95	.39	.91	.89

**Notes.**

* Indicates that a correlation is significant at a level of *p* < 0.05 (two sided).

** Indicates that a correlation is significant at a level of *p* < 0.01 (two sided).

a Deleting the item with poor load or splitting into search- and memory-tasks did not lead to significant correlations with depression.

PHQ Patient Health Questionnaire Web-CLIC Website –Clarity, Likeability, Informativeness, Credibility VisAWI Visual Aesthetics of Websites Inventory

Comparing the ratings (see Tables 3 and 4 for M and SD) for website A (actually existing) and B (mock-up website), there were no significant differences concerning the ratings of content (*t* =  − 1.66, *p* = .10), nor the reported intention to revisit (*t* =  − 1.79, *p* = .08). We found significant differences with regard to subjective usability (*t* = 4.71, *p* < .01) and aesthetics (*t* =  − 5.76, *p* < .01): while the mock-up website was evaluated to be more usable compared to the really existing website, it was perceived as less aesthetic.

**Table 4:** Study 2: mean, standard deviations and correlations among variables of interest (*N* = 392)—website B “MedOnline”.

Measure	1	2	3	4	5	6
1. Depression (PHQ)	–	−.08	−.09	−.06	−.10^*^	.03
2. Content (Web-CLIC)		–	.60^**^	.31^**^	.75^**^	.71^**^
3. Usability (subjective)			–	.28^**^	.57^**^	.44^**^
4. Usability (objective)				–	.25^**^	.19^**^
5. Aesthetics (VisAWI)					–	.56^**^
6. Intention to revisit						–
MW	7.70	4.65	4.80	3.79	4.46	3.11
SD	6.03	1.23	1.52	1.65	1.44	1.58
Range	0–27	1–7	1–7	0–7	1–7	1–7
Item load	.58–.88	.69–.87	.83–.96	.05^a^–.86	.77–.89	.81–.96
Cronbach’s Alpha	.90	.95	.96	.54	.90	.89

**Notes.**

* Indicates that a correlation is significant at a level of *p* < 0.05 (two sided).

** Indicates that a correlation is significant at a level of *p* < 0.01 (two sided).

a Deleting the item with poor load or splitting into search- and memory-tasks did not lead to significant correlations with depression.

PHQ Patient Health Questionnaire Web-CLIC Website—Clarity, Likeability, Informativeness, Credibility VisAWI Visual Aesthetics of Websites Inventory

**Figure 3:**
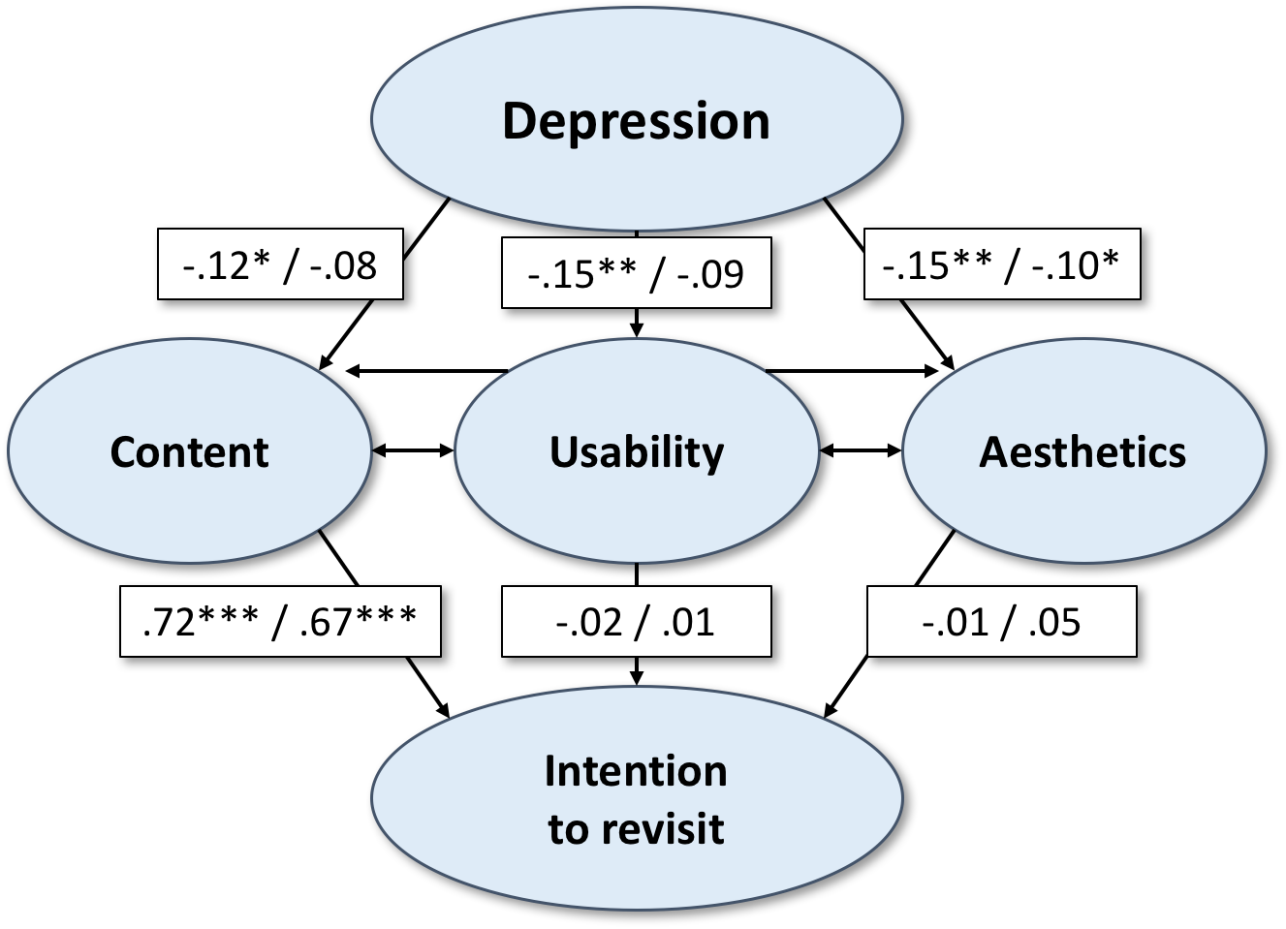
Structural equation model A and B. Structural model showing significant relationships between the variables of interest in study 2 A (“Physical training”—website with information on health exercises) and B (“MedOnline”—health information).

**Table 5:** Parameters (standardized).

Regression on/correlation with	A. Physical training			B. MedOnline		
	Estimate	S.E.	Two-tailed *P*-value	Estimate	S.E.	Two-tailed
Content on Depression	−0.12	0.05	0.02	−0.08	0.06	0.16
Usability on Depression	−0.15	0.05	<0.01	−0.09	0.05	0.09
Aesthetics on Depression	−0.15	0.06	<0.01	−0.10	0.05	0.04
Intention to Revisit on						
Content	0.72	0.06	<0.01	0.67	0.05	<0.01
Usability	−0.02	0.06	0.75	0.01	0.05	0.81
Aesthetics	−0.01	0.05	0.91	0.05	0.05	0.29
Content with						
Usability	0.69	0.03	<0.01	0.60	0.04	<0.01
Aesthetics	0.76	0.03	<0.01	0.75	0.02	<0.01
Usability with Aesthetics	0.70	0.03	<0.01	0.56	0.04	<0.01

**Table 6:** Model fit information.

Fit-Indice	Value	
	A. Physical training	B. MedOnline
Chi Quadrat		
Value	1.190	6.914
Degrees of freedom	1	1
*P*-value	0.275	0.009
RMSEA	0.022	0.123
CFI	1.000	0.993
TLI	0.998	0.929
SRMR	0.008	0.020

While the findings regarding website A (correlations and paths; see Fig. 3, Tables 5 and 6) resemble the findings in Study 1 (depression and UX: −.15 ≤ *β*’s ≤ − .12, all *p*’s <.05), the findings referring to website B slightly differ: the pathways leading from depression to content and usability do not emerge as significant (−.09 ≤ *β*’s ≤ − .08, .09 ≤ *p*’s ≤.16). However, the impact on aesthetics remains significant in A (*β* =  − .15, *p* < .01) and also in B (*β* =  − .10, *p* = .04). Likewise, the path leading from content to intention to revisit is significant in both cases (A: *β* = .69, *p* < .01; B: *β* = .67, *p* < .01).

On the whole, the hypothetical model fitted the data. As the chi-square test is considered very strict in modeling large sample sizes (and undesirably does become significant in B), alternative measures of approximate fit were added. As shown in Table 6, good fit is indicated for A (CFI = 1.000, TLI = 0.998 and RMSEA = 0.022, SRMR = 0.008), while the model fit (cf. 2.2) in B appears to be worse mostly with regard to RMSEA (CFI = 0.993, TLI = 0.929 and RMSEA = 0.123, SRMR = 0.020). Correspondingly, reducing the model (B) by the non-significant paths (cf. Fig. 3 and Table 5) leads to a strong improvement (*Chi-square* = 9.531, *df* = 4, *p* = .05) meeting a cut-off value close to .06 (RMSEA = 0.059) displaying good fit by the abridged model (Hu & Bentler, 1999).

### Discussion

Comparing the models regarding both websites in this study, we find again shared evidence for the pathway between depression and aesthetics (confirming *H3*), as well as for the one leading from content to the intention to revisit (confirming *H4*). The importance of the remaining paths we expect to be relevant due to theoretical considerations (from depression to content and from depression to usability) differs between websites: while the evaluation of the actually existing website (A) involves a biased perception of content (confirming *H1*) and usability (confirming *H2a*) due to depression, the mock-up site (B) partly prevented such a distortion and the path coefficient (rejecting *H1* and *H2a*) did not reach significance.

As we now find a significant correlational association of objective usability and the intention to revisit in both cases (A and B), we can assume that the operationalization of this website feature is improved. However, we still do not find significant correlational relationships between depression and performance as the measure of objective usability (rejecting *H2b*). The low medium score on the objective usability tasks indicates that items were not too easy to answer. Thus, objective usability seemed to be not notably impaired by depression severity, at least in the range analysed in the present studies.

## General Discussion

Taking together, the results obtained from Study 1 and 2, depressiveness emerges as capable to globally alter the subjective perception of a website as far as the user experience features content, usability, and aesthetics are concerned (confirming *H1, H2a, H3*). In contrast, common online tasks such as searching for certain information or recalling it after a short period of time do not seem to be altered by depression (rejecting *H2b*). Even though we do not find an impairment with regard to such objective performance scores, depression was associated with restrictions in the subjective usability evaluation (confirming *H2a*). As depression is lowering the evaluation of a website, the intention to revisit will be indirectly lowered as well.

When comparing the different website features, content seems to influence users’ intention to revisit a website in particular (confirming *H4*) while usability (rejecting *H5a, H5b*) and aesthetics (rejecting *H6*) do not. The latter finding might be due to the circumstance that we chose websites aimed at informing rather than entertaining. Another mechanism might be found regarding differences in neural processing, as central processing is assumed for a website’s content and rather peripheral processing for usability and aesthetics (Thielsch & Hirschfeld, in press). Therefore, considered decisions such as revisiting a website in the future might be more closely linked to content—while spontaneous reactions or an overall liking (see Thielsch, Blotenberg & Jaron, 2014) might show particular connections with usability and aesthetics.

### Practical implications

As the use of online (self-)help appears as a promising approach in altering depressive symptoms and avoiding barriers of traditional offline-treatment (e.g., Knowles et al., 2014; Richards & Richardson, 2012), the present studies’ results may help to prevent possible difficulties in designing such online environments. Content, usability and aesthetics will have to withstand a possibly biased evaluation given by people suffering from depression. Importantly, as we investigated the variables in general population, we might underestimate the strength of the (indeed small) effect—that may be more pronounced in a more severe depressed sample and that can only be observed in its entirety in a clinical sample.

As content appears to be crucial in deciding whether a website is visited again (which will be necessary to bring about a change), the first goal should be to improve comprehensibility, credibility and perceived benefit of a website. This could be achieved by extensively investigating the users’ expectations, fully responding to those, or possibly link together series of websites fulfilling the requirements as a whole. Furthermore, a website with controlled usability leading to superior subjective ratings (Study 2, mock-up website) antagonized the negative influence of depression on perceived usability. This attempt should be analysed more thoroughly to define helpful improvement suggestions for designing e-health websites. In the present study, the mock-up website was optimized in terms of an easy navigation and concise lists of relevant topics on first-level subpages.

Finally, our data show that depression is not necessarily influencing objective performance scores. Thus, in website evaluations with users it is important to take objective and as well subjective measures into account, as they seem to differ. Additional checklists or expert-based investigations of a website in question (see e.g., Hasan & Abuelrub, 2011; Prusti et al., 2012) might help to estimate the amount of bias in subjective evaluations of depressive users.

### Limitations and future research

Some limitations should be considered while interpreting the results of our studies, most of them directly point at avenues for future research.

Firstly, one has to keep in mind that we tested in the general population applying a web-based screening approach. Thus, there was no individual diagnostic procedure enclosing measures such as interviews. There is a potential bias in self-administered scales. Yet web-based survey methods are highly feasible for research as such bias is reduced, among other things due to participants perception of high anonymity and thus a lower social desirability bias (e.g., Crutzen & Göritz, 2010; Kreuter, Presser & Tourangeau, 2009; Pealer et al., 2001). Furthermore, investigating the general population is a basic step before testing in clinical samples and potentially burdening patients with extensive experimental setups (e.g., Krampen, Schui & Wiesenhütter, 2008). Yet a necessary and interesting next step would be to replicate the findings testing in clinical samples of people affected by MDD. An effect that can be observed in a non-clinical sample, where the phenomena of interest are less pronounced, is expected to be of relevance and more pronounced in a clinical sample.

Secondly, we found effects of demographics on outcome measures, in particular of age on usability (in Study 1) and on intention to revisit (in Study 2). The influence of age found in Study 1 is in line with prior research (Sonderegger, Schmutz & Sauer, 2016) and illustrates the general relevance of such user characteristics in user experience studies.

Thirdly, even though we extended the tested websites and replicated results in Study 2, the restricted range of stimuli, all dealing with topics related to e-health, does not allow drawing generally valid conclusions. Hence, further investigation of preferably diverging websites is needed. In addition, a huge amount of mobile phone depression apps is available (see Huguet et al., 2016; Shen et al., 2015), presenting another highly interesting field for user experience research.

Fourth, as we do not find substantial relationships between depression and objective usability, further investigation is needed to establish whether the effect might exist in severe depression or for other aspects of objective usability—or objective usability is not impaired by depression at all.

Finally, we focused on information websites and thus used the intention to revisit as important dependent variable. In clinical practice, online interventions are of high interest (Andersson & Cuijpers, 2009). In such studies one main variable of interest would be drop-out. Intentions are positively correlated to actual behavioural outcomes (e.g., Prestwich, Perugini & Hurling, 2008) and a positive user experience facilitates actual revisiting (Van ’t Riet, Crutzen & De Vries, 2010). Future research on health interventions online will benefit from a detailed analysis of web user experience, intentions, and actual revisiting (respectively drop-out). Thus, the present two-part study might be a valuable step towards the investigation of web user experience in the light of mental problems. In further studies, the exact mechanisms and pathways should be examined, to determine how depression as a symptom cluster exactly alters the web user experience (for example via sleep or concentration problems).

## Conclusion

Based on our findings, we assume that depressive symptoms affect individuals’ perception of websites and negatively alter their user experience. We present significant associations between depression and different web user experience features, as well as an indirect pathway from depression to the intention to revisit (as the perceived quality of content affects this intention). While the latter connection arises as strong in both studies, the impact from depression on user experience appears rather small (according to the guidelines provided by Cohen, 1988). However, as we do find the hypothetical connection in an unselected sample, an underestimation of the actual effect in a clinical sample can be assumed.

The found biased website impression should especially be considered when designing E-(Mental)-Health-platforms, which aim at providing information for users with mental disorders. Such online platforms are needed to improve the treatment situation by patronising treatment sections such as psycho-education or particular evidence-based interventions. Given the high prevalence of depressive symptoms in the population, such user characteristics might become relevant for general investigations of user experiences in fundamental and applied research as well, even as found effect sizes are small.

##  Supplemental Information

10.7717/peerj.4439/supp-1 Supplemental Information 1 Appendix

10.7717/peerj.4439/supp-2 Supplemental Information 2 Raw data for study 1 and study 2 To maintain confidentiality and privacy of participants, any demographic information as well as any information that might lead to the identification of participants were removed.

## Additional Information and Declarations

### Data Availability

The following information was supplied regarding data availability: The website evaluation data of both studies are provided as  Supplemental Files.
