# Supplementary material for: Depressive symptoms and web user experience
Source: PeerJ. 2018 Feb 28;6:e4439. doi: 10.7717/peerj.4439 (PMC5834939; doi:10.7717/peerj.4439)
Supplement: Supplemental Information 1 [file peerj-06-4439-s001.pdf]

Appendix

Table A.1. *Subscales and items of the Web-CLIC (Anonymized, in press) in English and German*

**Items English**	**Items German**
Clarity	*Verständlichkeit*
The contents of the website to be clearly arranged.	Die Inhalte sind anschaulich aufbereitet.
The texts provide me information in a clear and concise manner.	Die Texte liefern mir kurz und bündig die wichtigsten Informationen.
The language used in the texts to be current and easy to understand.	Der Sprachgebrauch in den Texten ist geläufig und allgemein verständlich.
Likeability	*Gefallen*
The website arouses my interest.	Die Website weckt mein Interesse.
The contents of the website are exciting.	Die Inhalte der Website sind spannend.
I enjoy reading the website.	Ich lese diese Website gerne.
Informativeness	*Informationsgehalt*
The information is of high quality.	Die Informationen sind qualitativ hochwertig.
I find the information on the website to be useful.	Ich finde die Informationen auf der Website nützlich.
The website is informative.	Die Website ist informativ.
Credibility	*Glaubwürdigkeit*
I find the information provided on the website to be authentic.	Die auf der Website dargebotenen Informationen sind glaubwürdig.
The information provided on the website to be reliable.	Die auf der Website dargebotenen Informationen sind seriös.
I can trust the information on the website.	Ich kann den Informationen auf der Website vertrauen.

Note. Scale: 7-point Likert scale ranging from 1 (“strongly disagree”) to 7 (“strongly agree”).

Table A.2. *All items of the PWU in English and German (original scale at Flavián et al., 2006; information on German adoption at Thielsch, 2008 and Thielsch et al., 2015).*

**Items English**	**Items German**
I think the use of this website is easy to understand.	Ich finde, die Bedienung der Website ist leicht zu verstehen.
This website is simple to use, even when using it for the first time.	Die Website ist einfach zu benutzen, sogar wenn sie zum ersten Mal besucht wird.
It is easy for me to find the sought information.	Es fällt mir leicht, die gesuchten Informationen zu finden.
I can easily understand the structure of this website.	Ich kann die Struktur der Website leicht nachvollziehen.
It is easy to navigate within this website.	Es ist einfach, sich auf der Website zurechtzufinden.
Contents are organized in a way that I know where I am at any time.	Die Inhalte sind so organisiert, dass ich jederzeit weiß, wo ich mich befinde.
I am able to find the required information quickly.	Ich kann gesuchte Informationen schnell erreichen.

Note. Scale: 7-point Likert scale ranging from 1 (“strongly disagree”) to 7 (“strongly agree”).

Table A.3. *Subscales and items of the VisAWI-S (Moshagen & Thielsch, 2013) in English and German*

**Items English**	**Items German**
Everything goes together on this site.	Auf der Seite passt alles zusammen.
The layout is pleasantly varied.	Das Layout ist angenehm vielseitig.
The color composition is attractive.	Die farbliche Gesamtgestaltung wirkt attraktiv.
The layout appears professionally designed.	Das Layout ist professionell.

Note. Scale: 7-point Likert scale ranging from 1 (“strongly disagree”) to 7 (“strongly agree”).

Table A.4. Item difficulty for search – and memory tasks used in study 1 (website „depression“)

Type of task	Item	Item difficulty
Search tasks	How many patients profit from antidepressants in the first two weeks of treatment? [two thirds; three quarters; one half; despite an intensive search I could not find the requested information]<br>*Bei wie vielen Patienten bessern sich die Depressionssymptome innerhalb der ersten zwei Wochen der medikamentösen Therapie mit Antidepressiva? [Bei zwei Dritteln; Bei drei Vierteln; Bei der Hälfte; Bei einem Drittel; Trotz intensiver Suche habe ich die Information nicht gefunden.]*	.72
	How many symptoms have to persist for how long to correctly diagnose a major depressive disorder? [two main and two minor symptoms for two weeks; one main and three additional symptoms for two weeks; six symptoms for two weeks and one out of them has to be a major one; despite an intensive search I could not find the requested information]<br>*Wie viele Symptome müssen wie lange bestehen, um die Diagnose einer Depression stellen zu können? [Zwei sog. Hauptsymptome und zwei Nebensymptome über zwei Wochen; Ein Haupt- und drei Nebensymptome über zwei Wochen; Mind. sechs Symptome über zwei Wochen, von denen eins ein Hauptsymptom sein muss; Zwei Haupt- und ein Nebensymptom über zwei Wochen; Trotz intensiver Suche habe ich die Information nicht gefunden.]*	.73
Memory tasks	What percentage of the people older than 65 years are suffering from depression? [ca. 10%; ca. 5%; ca. 1%; ca. 30%]<br>*Wie viel Prozent (%) der Über-65-jährigen leiden an einer Depression? [Ca. 10%. Ca. 5%. Ca. 1%. Ca. 30%]*	.19
	What is the real amount of victims with a depressive disorder that can be estimated according to WHO? [ca. 20% of the affected; ca. one third of the affected; ca. 40% of the affected; ca. half of the affected]<br>*Wie hoch ist laut WHO die Dunkelziffer bei Depressionen? [Ca. 20% der Betroffenen; Ca. ein Drittel der Betroffenen; Ca. 40% der Betroffenen; Ca. die Hälfte der Betroffenen]*	.38
	Which illness can cause depressive symptoms? [Gout; Diabetes mellitus; Rheumatism; Cataract]<br>*Welche Krankheit kann oftmals der Auslöser für eine depressive Symptomatik sein? [Gicht; Diabetes Mellitus; Rheuma; Grauer Star]*	.75
	Why does depression often not get treated in the elderly? [poor prospects of success; problems with health insurance company's coverage; depression is diagnosed late; relatives disapprove attempts to change and	.88

therefore boycott therapy]<br>*Warum werden Depressionen im Alter oft nicht behandelt? [Wenig Aussicht auf Therapie-Erfolg; Schwierigkeiten mit der Kostenübernahme durch Krankenkassen; Sie werden zu spät erkannt; Angehörige akzeptieren keine Veränderung mehr der betroffenen Person und behindern die Therapie]*	
How often and how long does one have to be outdoors in order to produce enough vitamines and prevent seasonal distortion and depression? [half an hour every day; a quarter of an hour every day; a quarter of an hour every other day; an hour every three days]<br>*Wie oft und lange muss man nach draußen gehen, um genug Vitamine in der Haut zu bilden und einem Herbst-Winter-Blues vorzubeugen? [Jeden Tag eine halbe Stunde; Jeden Tag eine Viertelstunde; Alle 2 Tage eine Viertelstunde; Alle 3 Tage eine Stunde]*	.39

Note. Original German wording displayed in blue.

Figure A.1. Screenshot example from study 2; mock-up website “Medonline”

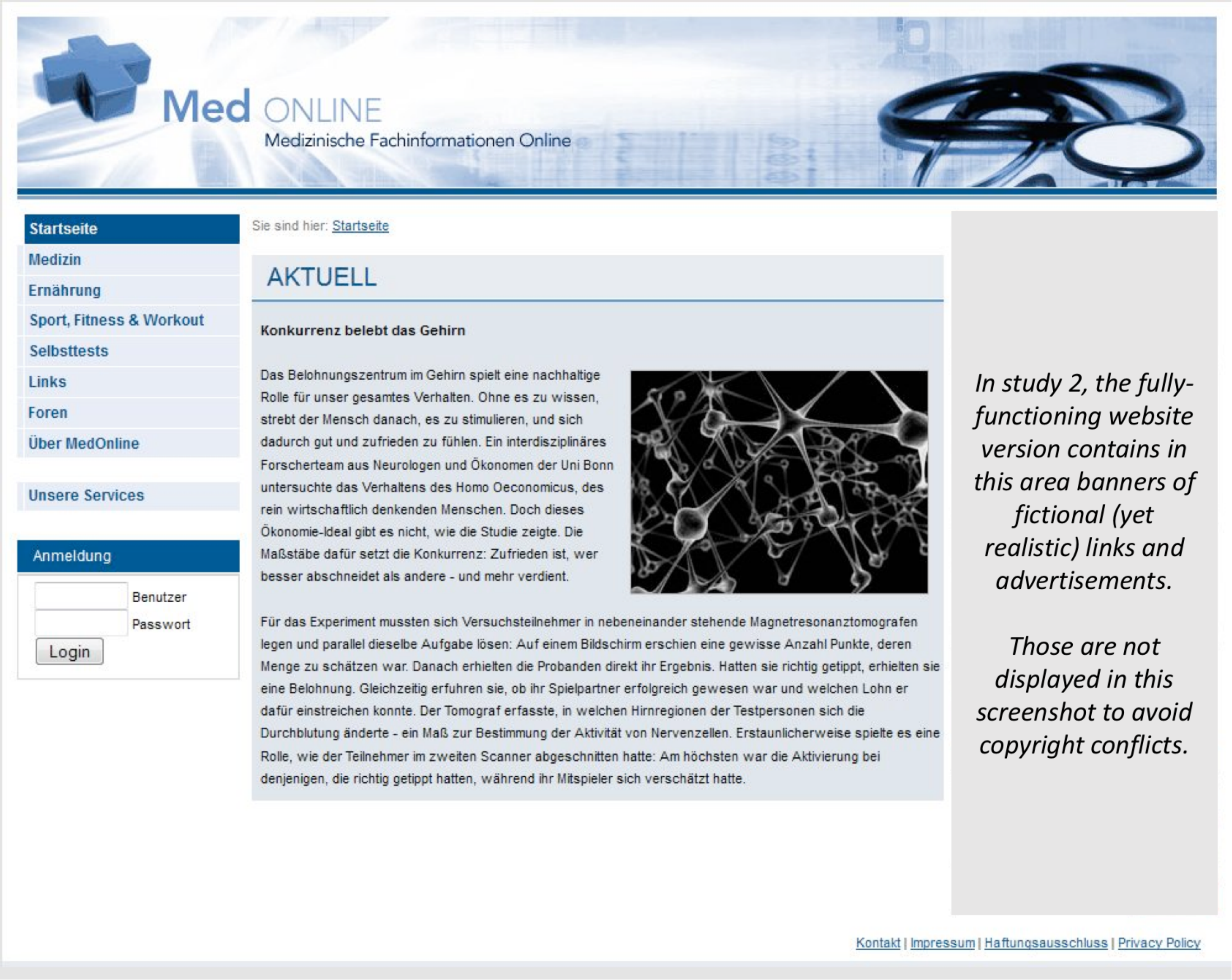

Table A.5. Item difficulty for search – and memory tasks used in study 2 (websites „physical training“ and „mock-up“)

Type of task	Website	Item	Item difficulty
Search tasks	A “physical training”	If you continue physical training up to old age, you can keep the average performance level of a person of what age? [free answer format]<br>*Wer bis ins Alter seine Ausdauer trainiert, kann das Leistungsniveau einer Person welchen Alters aufrechterhalten? [offenes Antwortformat]*	.83
		What problem occurs in many traditional strength exercises? [free answer format]<br>*Welches Problem gibt es bei vielen traditionellen Kraftübungen? [offenes Antwortformat]*	.27
		What do bob or ski-racer do just before the race? [free answer format]<br>*Was machen Bob- oder Skirennfahrer vor dem Rennen? [offenes Antwortformat]*	.19
	B “mock-up”	What percentage of the general population is suffering from glaucoma? [free answer format]<br>*Wie viel Prozent der Bevölkerung leiden schätzungsweise an einem Glaukom? [offenes Antwortformat]*	.76
		Which disorders do count as color-vision disorders and which number do affected people see on the test table? [free answer format]<br>*Welche Störungen zählen zu den Farbsehstörungen und welche Zahl erkennen farben-blinde Menschen auf der Farbtafel, mit der diese Farbsehstörungen untersucht werden? [offenes Antwortformat]*	.44
		What is arthrosis and which treatment can slow down the progress but not completely stop it? [free answer format]<br>*Was versteht man unter Arthrose und welche Maßnahme wird genannt, um Arthrose zu verzögern, aber nicht endgültig zu therapieren? [offenes Antwortformat]*	.27
Memory tasks	A “physical training”	What is the essential point in strength exercises? [to train variedly and stretch the muscles after every exercise unit; balanced nutrition to support muscle formation; strict control of success; correct exertion of strength exercises; to train those muscles specifically that are required daily; I do not know the correct answer]<br>*Was ist das A und O beim Krafttraining? [Antworten: vielseitig trainieren und die gekräftigte Muskulatur nach jeder Übungseinheit dehnen; ausgewogene Ernährung um die Muskelbildung zu Unterstützen; strenge Erfolgskontrolle; korrekte Ausübung der Kraftübungen; gezielt die Muskelpartien trainieren die im Alltag benötigt werden; weiß ich nicht]*	.41
		What proportion of all muscles in the body has to be exerted so that the specific type of sport is suitable as	.12

	endurance training? [a quarter, one fifth, a sixth, a seventh, an eighth; I do not know the correct answer]<br>*Wie viel der Körpermuskulatur muss beim Sport beansprucht werden damit sich die entsprechende Sportart zum Ausdauertraining eignet? [Antworten: 1/4; 1/5; 1/6; 1/7; 1/8; weiß ich nicht]*	
	Where has the companies' logo been positioned? [top left; in the middle on the right; in the middle on the left; in the centre**;** I do not know the correct answer]<br>*Wo war das Logo der Techniker Krankenkasse platziert? [Antworten: oben links; rechts mittig; links mittig; oben rechts; in der Mitte; weiß ich nicht]*	.76
	Which category was not listed under the group topic „physical training“? [fitness training; endurance training; movement training; strength training; mental training; I do not know the correct answer]<br>*Welche Rubrik war im Menü unter dem Überthema Training nicht aufgeführt? [Antworten: Fitnesstraining; Ausdauertraining; Bewegungstraining; Krafttraining; Mentaltraining; weiß ich nicht]*	.37
B "mock-up"	How is arthrosis called in colloquial language? [bone fragility; bone abrasion; bone erosion; wear of the joint; articulation fragility, I do not know the correct answer]<br>*Wie lautet der umgangssprachliche Begriff für das Wort Arthrose? [Antworten: Knochenbrüchigkeit; Knochenabnutzung; Knochenverschleiß; Gelenkverschleiß; Gelenkbrüchigkeit; Weiß ich nicht]*	.87
	Which numbers do people not affected with color blindness see on the test table? [162; 128; 182; 186; 216; I do not know the correct answer]<br>*Welche Zahlen können Menschen, die nicht farbenblind sind, auf der Farbtafel erkennen, die auf der Website zu sehen war? [Antworten: 162; 128; 182; 186; 216; weiß ich nicht]*	.49
	Where has the companies' logo been positioned? [top left; in the middle on the right; in the middle on the left; in the centre**;** I do not know the correct answer]<br>*Wo war das Logo von MedOnline platziert? (GF1, mittel) [Antworten: oben links; rechts mittig; links mittig; oben rechts; in der Mitte; weiß ich nicht]*	.58
	What was depicted in the picture on the right next to the article on the start website? [the human brain; a cross; a stethoscope; connected nerve cells; a magnetic resonance scanner; I do not know the correct answer]<br>*Depicted Was war auf dem Bild, das rechts neben dem Artikel auf der Startseite abgebildet war, zu sehen? [Antworten: das Gehirn eines Menschen; ein Kreuz; ein Stethoskop; Nervenzellen, die miteinander verbunden sind; ein Magnetresonanztomograph; weiß ich nicht]*	.39

Note. Original German wording displayed in blue.
